# The Use of Exercise Prescription in Australian Osteopathy Practice: Secondary Analysis of a Nationally Representative Sample of the Profession

**DOI:** 10.1155/2024/1977684

**Published:** 2024-06-14

**Authors:** Michael Fleischmann, Brett Vaughan, Kylie Fitzgerald

**Affiliations:** ^1^School of Health and Biomedical Science, RMIT University, Melbourne, Australia; ^2^Osteopathy College of Sport, Health and Engineering (CoSHE), Victoria University, Melbourne, Australia; ^3^Department of Medical Education, University of Melbourne, Melbourne, Australia

## Abstract

**Introduction:**

Exercise is beneficial for improving general health, wellbeing, and specific medical conditions. In musculoskeletal conditions such as chronic low back and neck pain, prescribed exercise has been found to be moderately effective in decreasing pain and improving function. Osteopaths are primary contact health professionals who manage predominantly musculoskeletal complaints. This work presents a secondary data analysis of the Australian osteopathy practice-based research network and profiles the characteristics of osteopaths who often use exercise prescription in patient care. *Methodology*. Secondary analysis of a cross-sectional survey of 992 osteopaths was registered with the Osteopathy Research and Innovation Network, an Australian practice-based research network. Demographics, practice, and treatment characteristics of Australian osteopaths who “often” use exercise prescription in patient care were examined.

**Results:**

Seven-hundred and thirty-three Australian osteopaths (74%) indicated that they use exercise prescription “often” in patient care. Australian osteopaths who often use exercise prescription are more likely to be colocated with another osteopath (ORa 1.54) and send referrals to an exercise physiologist (ORa 1.94) and a specialist medical practitioner (ORa 1.72). Those osteopaths who often used exercise prescription were also more likely to discuss physical activity (ORa 5.61) and nutrition (ORa 1.90). Australian osteopaths who use exercise prescription often were more likely to treat patients with sports injuries (ORa 2.43) and use soft tissue techniques (ORa 1.92), trigger point techniques (ORa 2.72), and sports taping (ORa 1.78).

**Conclusion:**

Osteopaths who utilise exercise prescription were more likely to discuss physical activity, diet, and nutrition and utilise referral networks with specialist medical practitioners and exercise physiologists. Australian osteopaths who often use exercise prescriptions were also more likely to treat patients with sport injury. The results suggest that most Australian osteopaths use exercise prescription and have referral networks with other health professionals for patient management. Further work is required to explore the type of exercise prescription used and for what conditions.

## 1. Introduction

Research highlights the benefits of exercise including improving general health and wellbeing and being part of the management of specific medical conditions such as stroke and osteoporosis [1]. Although general exercise is beneficial for an individual's quality of life [2] and from a public health perspective [3], more specific or targeted exercise can be of benefit in the management of various complaints, particularly those that are musculoskeletal in nature. For chronic low back pain (LBP) and neck pain, prescribed exercise has been demonstrated to be moderately effective in decreasing pain and improving function [4, 5]. Prescribing exercise within management plans for musculoskeletal conditions is strongly advocated in both clinical practice guidelines [6–9] and biopsychosocial approaches to patient care [10].

There are a range of prescribed exercise approaches that can be utilised as part of the management of musculoskeletal complaints such as low back pain. These include resistance training, aerobic-based exercise, motor control exercises, and other exercise approaches including yoga and Pilates [11]. Walking [12] and aquatic-based exercise [13] are also reported to be of benefit as an exercise for musculoskeletal complaints. However, a significant challenge with the inclusion of exercise as part of the management plan is adherence, both from a patient and research definition perspective [14]. That said, these exercise interventions are relatively easily incorporated into a management plan based on patient preference(s) and beneficial from a public health and health promotion perspective.

Exercise prescription is frequently used by allied health professionals with the aim of improving physiological well-being, functional ability, capacity, mobility, and pain relief [15–17]. These allied healthcare professions include physiotherapy [18], chiropractic [18, 19], occupational therapy [20], and osteopathy [21]. The type of exercise prescription provided typically includes activity recommendations, progressive general exercise, and more specific exercise interventions including stretching, range of motion activities, and stabilisation exercises to specific body regions [22].

Osteopaths are primary contact health professionals who manage predominantly musculoskeletal complaints [23]. In the Australian context, patients predominantly access osteopathy services privately. Patients may also access care through third-party payment schemes associated with worker's compensation, traffic accident, and war veteran schemes. Although the dominant therapeutic approach used by Australian osteopaths is manual therapy [24], patient education [25, 26] and exercise are also incorporated. Orrock [27] explored osteopathic practice in Australia in 2009 and found that approximately 55% of practitioners often or always prescribe therapeutic exercise. In 2018, Adams, Sibbritt, Steel, and Peng [23] reported 74% of Australian osteopaths utilise exercise prescription, as do 78% of New Zealand osteopaths. These increases over time in the reported use of exercise prescription may be related to an increase in the visibility of this approach within osteopathy pre-professional curricula.

Data from the United Kingdom (UK) show that approximately 23% of osteopaths used exercise prescription as part of their patient management [28]. In contrast, a cross-sectional study of Australian osteopaths in 2013 reported approximately 6% only of patient records examined in 2011 and 2012 contained a form of exercise prescription [29]. As highlighted above, the lower rate in this older work by Burke, Myers, and Zhang [29] compared to more contemporary research reflects an increasing utilisation in practice, likely based on the education of osteopaths and emphasis on exercise as an effective management strategy for musculoskeletal complaints.

Several case studies have also reported osteopaths prescribing exercise as a form of therapy or where it has been incorporated into the patients' broader management plan [30–32]. The use of exercise outside the immediate osteopathy practice environment has also been investigated with home exercise programmes featured in various manual therapy research studies [33]. However, there is limited higher quality research about the use of exercise prescription by osteopaths in the literature, suggesting that further research is needed to effectively capture the use of exercise prescription by the profession. Our work presents a secondary data analysis of the Australian osteopathy practice-based research network [23, 24] to profile the characteristics of osteopaths who often use exercise prescriptions in patient care [34]. In profiling the practice of Australian osteopaths and their use of exercise, it is possible to develop a more informed understanding of which practitioners are using exercise prescription in practice. Furthermore, the current analysis also allows for an initial exploration as to whether the practice of Australian osteopaths reflects contemporary evidence-based approaches using combined manual therapy and exercise prescription for the management of musculoskeletal complaints.

## 2. Methods

### 2.1. Participants

Ethics approval for the data collection was granted by the University of Technology, Sydney, and Human Ethics Committee (# 2014000759). The Australian Osteopathy Practice-Based Research Network (PBRN) as part of the Osteopathy Research and Innovation Network (ORION) project [23] was used to recruit participants from July to December 2016. Potential participants were required to be a registered osteopath at the time of data collection. Participants who consented were invited to complete an online questionnaire. Responses to the ORION questionnaire were received from 992 osteopaths, a 49.1% response rate. Adams and colleagues [23] reported the respondents to be nationally representative of the Australian osteopathy profession at the time of data collection.

### 2.2. Questionnaire

A 27-item questionnaire was developed to collect data from the PRBN participants using dichotomous, frequency, and Likert-type responses [23]. The questionnaire invited participants to provide data on individual practitioner demographics (i.e., age, gender, and number of years in private osteopathy practice), participants' practice characteristics (i.e., patient care hours and patient visits per week, practice location, and interactions with other health professionals either through colocation or referrals), and patient management (i.e., body regions treated, manual therapy technique use, and advice to patients). Additional items also explored practitioner opinions on expanded practice rights and use of research in osteopathy practice. Patient management characteristics included the frequency of patient presentations, discussion of lifestyle behaviors, frequency of treating specific patient groups, and frequency of osteopathy technique use.

### 2.3. Outcome Variables and Exposure Variables

Participants were asked to indicate their frequency of use of exercise prescription in patient care (“never,” “rarely,” “sometimes,” and “often”), the outcome variable. The outcome variable was dichotomized to “not often” (combining never, rarely, and sometimes) or “often” [35]. The exposure variables were the practitioner and practice characteristics described in the Questionnaire section above. Variables with frequency or Likert-type responses were dichotomized for the analysis (*often* and *not often* (“never,” “rarely,” and “sometimes”)) and attitude (*definitely* and *not definitely* (“no,” “unsure,” “maybe”)). Age, average patient numbers per week, average patient care hours per week, and years in clinical practice were analysed as continuous variables. All other variables included in our analysis are reported in binary form (yes/no).

### 2.4. Statistical Analyses

Analyses were performed using SPSS (version 25). Descriptive statistics were generated for each variable on the questionnaire. Inferential statistics were used to explore the association between the outcome variable and dichotomized variables. Alpha was set at *p* < 0.05, and unadjusted odds ratios ORc (with 95% confidence intervals) calculated where significant. Continuous data were analysed using independent measures *t*-tests with alpha set at *p* < 0.05, and effects sizes (Cohen's *d*) calculated where significant. Variables with *p* < 0.20 were entered into a binary logistic regression analysis. Backward elimination was used to determine the significant predictors of osteopaths who “often” use exercise prescriptions [23]. Adjusted odds ratios (ORa) with 95% confidence intervals (CI) and *p* values were calculated from this regression modelling. Variables were significantly associated with the outcome variable at *p* < 0.05.

## 3. Results

Seven-hundred and thirty-three Australian osteopaths (73.9%) indicated that they use exercise prescription “often” in patient care. There was no statistically significant difference of gender for Australian osteopaths who use exercise prescription often compared to osteopaths who do not use it often (*p* > 0.05) (Table 1). Australian osteopaths who often use exercise prescription were younger in both age and time in practice (*p* < 0.05) and reported a higher number of patient visits and care hours per week (*p* < 0.05) all with small-to-medium effect sizes. Those Australian osteopaths with a postgraduate qualification, and those who reported being a member of Sports Medicine Australia, were also more likely to use exercise prescription often, compared to those who did not report these characteristics (Table 1).

For patient assessment, Australian osteopaths who use exercise prescription often were more than twice as likely to refer for diagnostic imaging and six times more likely to use orthopaedic assessment in patient examination, compared to those who do not often use exercise prescription (Table 2). Australian osteopaths who often use exercise prescription were approximately 50% more likely to be colocated with other osteopaths (ORc 1.48) and nearly twice as likely to send referrals to exercise physiologists (ORc 1.90) (Table 2).

Australian osteopaths who often use exercise prescription in patient care were more than eight times as likely to discuss physical activity with their patients compared with osteopaths who do not often use exercise prescription (Table 3). Medication and occupational health and safety were more than twice as likely to be discussed with patients by osteopaths who reported the use of exercise prescription often in patient care (Table 3). Australian osteopaths who often use exercise prescription were almost twice as likely to discuss a range of other clinical management strategies with patients compared with osteopaths who do not often use exercise prescription (Table 3).

Osteopaths who often use exercise prescription were more than twice as likely to treat postural disorders (ORc 2.13) and tendinopathies (ORc 2.28) and compared to those who do not often use exercise prescription in patient care (Table 3). Australian osteopaths who often use exercise prescription were three times more likely to treat patients with sport injuries (ORc 3.37) and twice as likely to report treating compensable work injury patients (ORc 2.40) (Table 3).

Osteopaths who often used exercise prescription were more than twice as likely to use muscle energy technique and dry needling and three times more likely to more than 3x more likely to use soft tissue technique and trigger point therapy (Table 3). Those osteopaths who often used exercise prescription were also nearly six times more likely to use sports taping compared with colleagues who did not often use exercise prescription (Table 3). However, osteopaths who often use exercise prescription were less likely to use autonomic balancing, balanced ligamentous tension, biodynamics, and osteopathy in the cranial field techniques in patient care (Table 3).

Australian osteopaths who often use exercise prescription in patient care were nearly twice as likely to indicate expanded practice with respect to prescribing rights (ORc 1.92) and twice as likely to seek expanded referral rights to Sports Medicine specialists (ORc 2.37) (Table 3).

Adjusted odds ratios (ORa) for variables that were identified as being statistically significant in the backward binary logistic regression model are described in Table 4. Australian osteopaths who *often* use exercise prescription were over five times more likely to discuss physical activity with patients compared with those who do *not often* use exercise prescription in patient care.

## 4. Discussion

Our secondary analysis of the Australian osteopathy PBRN data provides a novel insight into the practice characteristics of practitioners who often use exercise prescription as part of the care of patients with musculoskeletal complaints. Approximately three-quarters of Australian osteopaths often prescribe exercise in patient care. This finding is consistent with previous data for New Zealand osteopaths [24] suggesting that exercise prescription is a significant component of Australasian osteopathy practice.

Our data show that Australian osteopaths who often use exercise prescriptions are also more likely to engage in referrals with other health professionals. Osteopaths who use exercise prescription often were almost twice as likely to send referrals to an exercise physiologist and to specialist medical practitioners. Approximately 5% of referrals from Australian osteopaths are reported to be specialist medical practitioners [27]. These findings are encouraging, and it may indicate that these osteopaths are more likely to use a multidisciplinary approach to their patient management, particularly where exercise prescription is involved. It is possible that Australian osteopaths are working with exercise physiologists for individual patient care. There are opportunities for these two health professional groups to work together through the Australian government-funded chronic disease management plan scheme [36] and also in the care of private paying patients. Combined with the current findings, there is an increasing evidence base with respect to referrals to and from osteopaths [27, 29]. We are not able to comment on the nature of the referrals; however, these findings warrant additional exploration to better understand how Australian osteopaths work with other health professionals for the benefit of their patients.

The practice of osteopathy intersects with exercise and physical activity and well-being from several perspectives. Australian osteopaths who often use exercise prescriptions in patient care were over five times more likely to report discussing physical activity with their patients compared with osteopaths who do not. Our results suggest that osteopaths who discuss physical activity and use of exercise prescription form a significant part of Australian osteopathic practice [26]. This result is encouraging from a health promotion perspective and may indicate that Australian osteopaths, who incorporate exercise prescription into patient care, also recognise the value of physical activity for overall health. Activities such as walking and swimming are examples of physical activity that are beneficial for managing musculoskeletal complaints [12, 37], and it may be that osteopaths are encouraging patients to engage in these or other forms of physical activity. Further, these findings suggest that osteopaths may be playing an important role in promoting public health messaging around physical activity for general health. However, these assertions require further research through practice audits or practitioner interviews.

Our data suggest that osteopaths who report often using exercise prescription were more than twice as likely to treat sports injuries and 50% more likely to use sports taping. Injuries related to sport are common presentations to Australian osteopaths with approximately half of Australian osteopaths treating sport-related injuries [23]. The association observed between using exercise prescription and treating sport-related injuries is likely related to Australian osteopaths using targeted and sport-specific exercise to facilitate an individuals' return to sport post-injury [38]. However, exercise prescription for sports injuries in the context of osteopathy care is underexplored. Several case studies [39, 40] have described the use of exercise prescription in the context of osteopaths managing sport-related injuries, with positive outcomes. However, there are also opportunities to develop higher level evidence to support patient outcomes and cost-effectiveness. With respect to the use of sports taping, there is evidence to support its use for the management of musculoskeletal complaints, particularly in short term or for acute presentations [41–43]. Sports taping was also used by Australian osteopaths, and the association in the current works suggests that this forms part of their management exercise prescription for patient care. There is an evidence base for the use of taping for the management of a wide variety of musculoskeletal complaints [44] or to support athletic performance [45]. It may be that Australian osteopaths recognise the value of sports taping, combined with exercise prescription, for patient care. Further, the increased likelihood of sports taping use by osteopaths who often use exercise prescription suggests they may be combining these modalities in patient care; however, more exploration is needed.

Nutritional supplement advice was also more likely to be used by Australian osteopaths who often use exercise prescriptions, compared to those who do not. This is a consistent finding with the chiropractic profession [46] suggesting that this advice forms part of the scope of manual therapy practice in Australia. From a health promotion perspective, few Australian adults meet the fruit and vegetable intake guidelines [47], instead consuming a dominance of excessive calorie dense, ultraprocessed food intake, posing a risk for heart disease, type 2 diabetes, and several cancers [48]. The nature of the nutritional supplement advice provided by Australian osteopaths requires exploration, particularly whether this advice relates to specific supplements for the management of musculoskeletal complaints or is more broadly applicable to overall health and wellbeing.

Previous research has shown that a variety of manual therapy techniques are the dominant intervention strategy for Australian osteopaths [23, 27, 29]. Although the use of manual therapy by Australian osteopaths is common [23, 27, 29], our work highlights some techniques (soft tissue techniques and trigger point therapy) that are more commonly utilised by osteopaths who often use exercise prescription compared to those who do not. Soft tissue techniques have been shown to be effective in the management of upper and lower extremity musculoskeletal complaints [49–51]. As such, the use of soft tissue techniques by Australian osteopaths may facilitate the ability of a patient to undertake their prescribed exercises or potentially related to patient expectation with respect to the interventions provided.

The cross-sectional and self-report nature of the design of the ORION survey is a limitation when interpreting the results of the study. It is known that cross-sectional self-report designs are potentially susceptible to social desirability bias [52] and recall bias [53]. How practitioners defined exercise prescription when completing the questionnaire is open to interpretation and may have skewed the results. Lastly, the design of the survey does not allow for analysis of the type of exercise prescription (e.g., whether in the clinic or home) and whether osteopaths use exercise prescription for some presenting complaints only. It is probable that practitioners approach different conditions in different ways and this clinical reasoning would be valuable to explore.

Our analyses offer opportunities for future research to develop a greater understanding of how Australian osteopaths use exercise prescription in their practice. Barriers and enablers for the use of exercise prescription, the type of exercises being prescribed and for what presenting complaints, as well as the clinical reasoning for exercise prescription and outcomes from care where exercise prescription forms part of the management, could be explored. This research, combined with the current work, has the potential to inform pre- and postprofessional education (including professional development) and health policy.

## 5. Conclusion

Our work sought to identify the prevalence of exercise prescription used by osteopaths for patient management and to profile the clinical management characteristics of osteopaths who often use it. This work from a nationally representative practice-based research network (PBRN) profiles the characteristics of the 74% of Australian osteopaths who often use exercise prescription in patient management. We identified several patients and clinical management characteristics associated with the use of exercise prescription often in osteopathy patient care. These included discussion of physical activity, diet, and nutrition, often treating patients with sports injuries, and use of health professional referral networks. Whether these strategies are consistent with the best available evidence requires additional investigation, but the results support the conclusion that a significant proportion of Australian osteopaths often use exercise prescription in patient care.

## Figures and Tables

**Table 1:** Practitioner characteristics of Australian osteopaths who *often* versus *not often* use exercise prescription in patient care.

	Not often (*n* = 257)	Often (*n* = 733)	*p* value	OR [95% CI]
Gender				
Male	155 (15.7%)	420 (42.4%)	0.40	—
Female	102 (10.3%)	313 (31.6%)		
Age (years)				
Mean (±SD)	40.5 (±10.8)	37.1 (±10.7)	<0.01^a^	—
Years in clinical practice				
Mean (±SD)	13.6 (±9.0)	10.6 (±8.9)	<0.01^b^	—
Patient care hours per week				
Mean (±SD)	26.0 (±12.1)	28.6 (±12.0)	<0.01^c^	—
Patient visits per week				
Mean (±SD)	33.4 (±18.2)	37.5 (±18.6)	<0.01^d^	—
Qualification (*n*, %)				
Diploma	21 (2.1%)	41 (4.1%)		—
Advanced diploma	5 (0.5%)	4 (0.4%)		
Bachelor's degree	83 (8.4%)	134 (13.5%)		
Master's degree	141 (14.2%)	539 (54.4%)	<0.01	
PhD	2 (0.2%)	3 (0.3%)		
Other	5 (0.5%)	12 (1.2%)		
Involved in as an osteopath				
University teaching	34 (3.4%)	82 (8.3%)	0.38	—
Clinical supervision	40 (4.0%)	110 (11.1%)	0.83	—
Professional organisations	25 (2.5%)	82 (8.3%)	0.52	—
Research	14 (1.4%)	40 (4.0%)	0.99	—
Volunteer	40 (4.0%)	119 (12.0%)	0.80	—
Osteopathy Australia member	245 (24.7%)	701 (70.8%)	0.84	—
Sports Medicine Australia member	4 (0.4%)	50 (5.1%)	<0.01	4.63 [1.65, 12.95]
Chiropractic Australia member	9 (0.9%)	10 (1.0%)	0.03	0.38 [0.15, 0.95]

^a^ *d* = 0.31 95% CI [0.17, 0.45]; ^b^*d* = 0.33 95% CI [0.19-0.48]; ^c^*d* = 0.21 95% CI [0.07-0.35]; ^d^*d* = 0.22 95% CI [0.06, 0.38] (*d*: effect size).

**Table 2:** Practice characteristics of Australian osteopaths who often use exercise prescription in patient care.

	Not often	Often	*p* value	OR 95% [CI]
Practice location				
Urban practice	209 (21.1%)	610 (61.6%)	0.49	—
More than one practice location	64 (6.5%)	282 (28.5%)	<0.01	1.88 [1.37, 2.60]
Colocated with other health professionals (“yes”)				
Osteopath	149 (15.1%)	492 (49.7%)	<0.01	1.48 [1.10, 1.98]
General practitioner	13 (1.3%)	58 (5.9%)	0.13	—
Specialist medical practitioner	6 (0.6%)	25 (2.5%)	0.40	—
Podiatrist	36 (3.6%)	109 (11.0%)	0.73	—
Physiotherapist	25 (2.5%)	118 (11.9%)	0.01	1.78 [1.13, 2.81]
Exercise physiologist	16 (1.6%)	107 (10.8%)	<0.01	2.57 [1.49, 4.44]
Occupational Therapist	6 (0.6%)	13 (1.3%)	0.57	—
Psychologist	52 (5.3%)	138 (13.9%)	0.62	—
Massage Therapist	117 (11.8%)	382 (38.6%)	0.07	—
Acupuncturist	484 (4.8%)	139 (14.0%)	0.92	—
Naturopath	43 (4.3%)	150 (15.2%)	0.19	—
Dietician	16 (1.6%)	54 (5.5%)	0.54	—
Nutritionist	17 (1.7%)	60 (6.1%)	0.42	—
Send referrals to other health professionals (“yes”)				
Osteopath	127 (12.8%)	378 (38.2%)	0.55	—
General practitioner	224 (22.6%)	652 (65.9%)	0.43	—
Specialist medical practitioner	90 (9.1%)	353 (35.7%)	<0.01	1.72 [1.28, 2.31]
Podiatrist	149 (15.1%)	500 (50.5%)	<0.01	1.55 [1.16, 2.08]
Physiotherapist	85 (8.6%)	246 (24.8%)	0.88	—
Exercise physiologist	75 (7.6%)	322 (32.5%)	<0.01	1.90 [1.40, 2.58]
Occupational Therapist	28 (2.8%)	78 (7.9%)	0.91	—
Psychologist	99 (10.0%)	249 (25.2%)	0.19	—
Massage Therapist	170 (17.2%)	500 (50.5%)	0.54	—
Acupuncturist	125 (12.6%)	325 (32.8%)	0.23	—
Naturopath	130 (13.1%)	347 (35.1%)	0.37	—
Dietician	32 (3.2%)	133 (13.4%)	0.02	1.56 [1.03, 2.36]
Nutritionist	26 (2.6%)	102 (10.3%)	0.12	—
Receive referrals from other health professionals (“yes”)				
Osteopath	157 (15.9%)	456 (46.1%)	0.75	—
General practitioner	226 (22.8%)	658 (66.5%)	0.41	—
Specialist medical practitioner	47 (4.7%)	189 (19.1%)	0.01	1.55 [1.08, 2.22]
Podiatrist	105 (10.6%)	364 (36.8%)	0.01	1.42 [1.07, 1.90]
Physiotherapist	60 (6.1%)	205 (20.7%)	0.15	—
Exercise physiologist	52 (5.3%)	205 (20.7%)	0.15	—
Occupational Therapist	16 (1.6%)	45 (4.5%)	0.96	—
Psychologist	39 (3.9%)	115 (11.6%)	0.84	—
Massage Therapist	182 (18.4%)	570 (57.6%)	0.02	1.44 [1.04, 1.98]
Acupuncturist	102 (10.3%)	267 (27.0%)	0.35	—
Naturopath	92 (9.3%)	308 (31.1%)	0.08	—
Dietician	5 (0.5%)	33 (3.3%)	0.06	—
Nutritionist	12 (1.2%)	42 (4.2%)	0.52	—
Diagnostic imaging				
Referral for imaging (“often”)	10 (1.0%)	63 (6.4%)	0.01	2.32 [1.17, 4.60]
Investigation of unknown pathologies	176 (17.8%)	564 (57.0%)	<0.01	1.52 [1.12, 2.10]
Investigation of suspected diagnosis	216 (21.8%)	617 (62.3%)	0.96	—
Investigation of potential fractures	194 (19.6%)	554 (56.0%)	0.97	—
Rule out risk factors prior to treatment	67 (6.8%)	205 (20.7%)	0.56	—
General screening of the spine	7 (0.7%)	25 (2.5%)	0.38	—
Patient assessment (“yes”)				
Orthopaedic testing	241 (24.3%)	725 (73.2%)	<0.01	6.01 [2.54, 14.23]
Clinical assessment algorithm	95 (9.6%)	373 (37.7%)	<0.01	1.77 [1.32, 2.36]
Neurological testing	229 (23.1%)	687 (69.4%)	0.01	1.82 [1.11, 2.99]
Screening questionnaire	157 (15.9%)	476 (48.1%)	0.27	—
Cranial nerve testing	168 (17.0%)	502 (50.7%)	0.36	—

**Table 3:** Clinical management characteristics of Australian osteopaths who often use exercise prescription in patient care.

	Not high	High	*p* value	OR 95% [CI]
Discuss with patients (“often”)				
Diet	72 (7.3%)	303 (30.7%)	<0.01	1.82 [1.33, 2.45]
Smoking and drug use	31 (3.1%)	148 (59.1%)	<0.01	1.83 [1.21, 2.79]
Physical activity	185 (18.7%)	699 (70.7%)	<0.01	8.24 [5.29, 12.83]
Occupation health and safety	92 (9.3%)	412 (41.7%)	<0.01	2.32 [1.73, 3.12]
Pain counselling	80 (8.1%)	186 (18.8%)	0.07	—
Stress	111 (11.2%)	377 (38.2%)	0.02	1.39 [1.04, 1.85]
Nutrition	48 (4.9%)	203 (20.5%)	<0.01	1.67 [1.17, 2.38]
Medication	71 (7.2%)	319 (32.3%)	<0.01	2.03 [1.49, 2.76]
Patient presentations (“often”)				
Neck pain	251 (25.4%)	718 (72.6%)	0.68	—
Thoracic pain	233 (23.6%)	674 (68.1%)	0.48	—
Low back pain	253 (25.6%)	722 (73.1%)	0.69	—
Hip musculoskeletal pain	190 (19.2%)	552 (55.9%)	0.61	—
Knee musculoskeletal pain	115 (11.7%)	374 (37.9%)	0.08	—
Ankle musculoskeletal pain	79 (8.0%)	252 (25.5%)	0.29	—
Foot musculoskeletal pain	75 (7.6%)	217 (22.0%)	0.91	—
Shoulder musculoskeletal pain	195 (19.8%)	604 (61.2%)	0.02	1.48 [1.05, 2.10]
Elbow musculoskeletal pain	59 (6.0%)	190 (19.3%)	0.37	—
Wrist musculoskeletal pain	52 (5.3%)	136 (13.8%)	0.54	—
Hand musculoskeletal pain	36 (3.7%)	85 (8.6%)	0.29	—
Postural disorders	142 (14.4%)	531 (53.8%)	<0.01	2.13 [1.59, 2.86]
Degenerative spine conditions	128 (13.0%)	470 (47.6%)	<0.01	1.80 [1.35, 2.40]
Headache disorders	225 (22.8%)	665 (67.3%)	0.17	—
Migraine disorders	100 (10.1%)	299 (30.3%)	0.59	—
Spine health maintenance	97 (9.8%)	360 (36.5%)	<0.01	1.58 [1.18, 2.11]
Chronic or persistent pain	152 (15.4%)	476 (48.2%)	0.10	—
Tendinopathies	70 (7.1%)	338 (34.2%)	<0.01	2.28 [1.67, 3.12]
Temporomandibular joint disorders	39 (4.0%)	144 (14.6%)	0.12	—
Nonmusculoskeletal disorders	41 (4.2%)	85 (8.7%)	0.06	—
Patient subgroups (treat “often”)				
Up to 3 years of age	62 (6.3%)	94 (9.5%)	<0.01	0.46 [0.32, 0.66]
4 to 18 years of age	72 (7.3%)	196 (19.8%)	0.67	—
Over 65 years of age	134 (13.5%)	436 (44.1%)	0.04	1.38 [1.00, 1.78]
Aboriginal and Torres Strait Islander peoples	0	7 (0.7%)	0.11	—
Pregnancy	78 (7.9%)	265 (26.8%)	0.10	—
Non-English speaking	9 (0.9%)	24 (2.4%)	0.85	—
Sport injuries	74 (7.5%)	425 (43.0%)	<0.01	3.37 [2.48, 4.59]
Worker injury (compensable)	14 (1.4%)	89 (9.0%)	<0.01	2.40 [1.39, 4.29]
Work injury (noncompensable)	61 (6.2%)	280 (28.3%)	<0.01	1.97 [1.42, 2.73]
Traffic injury (compensable)	8 (0.8%)	46 (4.7%)	0.06	—
Traffic injury (noncompensable)	23 (2.3%)	91 (9.2%)	0.14	—
Postsurgery	14 (1.4%)	65 (6.6%)	0.09	—
Manual therapy (use “often”)				
Counterstrain	88 (8.9%)	331 (33.5%)	<0.01	1.58 [1.18, 2.13]
Muscle energy technique	173 (17.5%)	614 (62.0%)	<0.01	2.50 [1.80, 3.47]
High-velocity, low-amplitude manipulation	133 (13.4%)	498 (50.3%)	<0.01	1.97 [1.48, 2.64]
Joint manipulation	80 (8.1%)	312 (31.6%)	<0.01	1.63 [1.21, 2.21]
Soft tissue technique	188 (19.0%)	659 (66.6%)	<0.01	3.22 [2.23, 4.64]
Myofascial release	140 (14.2%)	472 (47.7%)	<0.01	1.50 [1.12, 2.00]
Visceral techniques	31 (3.1%)	67 (6.8%)	0.17	—
Lymphatic pump	23 (2.3%)	61 (6.2%)	0.75	—
Autonomic balancing	53 (5.4%)	104 (10.5%)	0.01	0.64 [0.44, 0.92]
Biodynamics	61 (6.2%)	94 (9.5%)	<0.01	0.47 [0.33, 0.68]
Functional technique	77 (7.8%)	193 (19.5%)	0.26	—
Balanced ligamentous tension	106 (10.7%)	243 (24.5%)	0.02	0.71 [0.52, 0.94]
Chapman's reflexes	2 (0.2%)	22 (2.2%)	0.06	—
Trigger point therapy	23 (2.3%)	234 (23.7%)	<0.01	4.78 [3.03, 7.54]
Osteopathy in the cranial field	93 (9.4%)	140 (14.2%)	<0.01	0.41 [0.30, 0.57]
Facilitated positional release	40 (4.0%)	126 (12.8%)	0.54	—
Dry needling	34 (3.4%)	200 (20.2%)	<0.01	2.47 [1.66, 3.66]
Shockwave therapy	3 (0.3%)	15 (1.5%)	0.36	—
Ultrasound	7 (0.7%)	20 (2.0%)	0.99	—
TENS	3 (0.3%)	16 (1.6%)	0.31	—
Instrument manipulation	0	2 (0.2%)	0.55	—
Instrument soft-tissue	2 (0.2%)	10 (1.0%)	0.36	—
Sport taping	8 (0.8%)	113 (11.4%)	<0.01	5.68 [2.73, 11.81]
Expanded practice scope (“definitely”)				
Prescribing rights	45 (4.5%)	212 (21.4%)	<0.01	1.92 [1.33, 2.74]
Referral rights to orthopaedic surgeon	158 (16.0%)	544 (54.9%)	<0.01	1.80 [1.33, 2.43]
Referral rights to paediatrician	131 (13.2%)	409 (41.3%)	0.18	—
Referral rights to sport and exercise medicine specialist	176 (17.8%)	613 (62.0%)	<0.01	2.37 [1.71, 3.92]
Referral rights to rheumatologist	149 (15.1%)	480 (48.5%)	0.03	1.37 [1.03, 1.84]
Referral rights to other medical specialist	0	1 (0.1%)	0.73	—
Expanded diagnostic imaging rights	194 (19.6%)	627 (63.3%)	<0.01	1.92 [1.35, 2.73]
Research (“strongly agree”)				
Help patients understand osteopathy	118 (11.9%)	324 (32.7%)	0.63	—
Help general practitioners and other health professionals understand osteopathy	168 (17.7%)	500 (52.6%)	0.48	—
Provide scientific evidence	130 (13.9%)	384 (41.0%)	0.68	—
Irrelevant to the development of osteopathy^*∗*^	141 (15.1%)	421 (45.0%)	0.49	—

^*∗*^ “strongly disagree.”

**Table 4:** Adjusted odds ratios for significant practitioners and clinical management characteristics of Australian osteopaths who *often* use exercise prescription in patient care.

	ORa	95% CI	*p* value
Years in practice	0.96	0.94, 0.98	0.002
Colocated with other osteopath (“yes”)	1.54	1.02, 2.31	0.038
Send referrals to an exercise physiologist (“yes”)	1.94	1.28, 2.94	0.002
Receive referrals from a naturopath (“yes”)	1.87	1.21, 2.88	0.005
Discuss physical activity (“often”)	5.61	3.11, 10.10	<0.01
Discuss nutrition (“often”)	1.90	1.13, 3.19	0.015
Treat postural disorders (“often”)	1.59	1.05, 2.40	0.026
Treat sports injuries (“often”)	2.43	1.61, 3.69	<0.01
Use soft tissue techniques (“often”)	1.92	1.14, 4.95	0.014
Use trigger point techniques (“often”)	2.72	1.49, 4.95	0.001
Use osteopathy in the cranial field (“often”)	0.47	0.29, 0.77	0.003
Use sports taping (“often”)	1.78	1.06, 2.98	0.041
Future prescribing rights (“definitely”)	1.79	1.06, 2.98	0.029

## Data Availability

Data are available on reasonable request by contacting sph_pbrn@uts.edu.au.
